# Novel insight into RNA modifications in tumor immunity: Promising targets to prevent tumor immune escape

**DOI:** 10.1016/j.xinn.2023.100452

**Published:** 2023-05-29

**Authors:** Yuxin Kong, Jie Yu, Shengfang Ge, Xianqun Fan

**Affiliations:** 1 Department of Ophthalmology, Shanghai Key Laboratory of Orbital Diseases and Ocular Oncology, Ninth People’s Hospital, Shanghai JiaoTong University School of Medicine, Shanghai 200001, China

## Abstract

An immunosuppressive state is a typical feature of the tumor microenvironment. Despite the dramatic success of immune checkpoint inhibitor (ICI) therapy in preventing tumor cell escape from immune surveillance, primary and acquired resistance have limited its clinical use. Notably, recent clinical trials have shown that epigenetic drugs can significantly improve the outcome of ICI therapy in various cancers, indicating the importance of epigenetic modifications in immune regulation of tumors. Recently, RNA modifications (N^6^-methyladenosine [m^6^A], N^1^-methyladenosine [m^1^A], 5-methylcytosine [m^5^C], etc.), novel hotspot areas of epigenetic research, have been shown to play crucial roles in protumor and antitumor immunity. In this review, we provide a comprehensive understanding of how m^6^A, m^1^A, and m^5^C function in tumor immunity by directly regulating different immune cells as well as indirectly regulating tumor cells through different mechanisms, including modulating the expression of immune checkpoints, inducing metabolic reprogramming, and affecting the secretion of immune-related factors. Finally, we discuss the current status of strategies targeting RNA modifications to prevent tumor immune escape, highlighting their potential.

## Public summary

• RNA modification is a novel hotspot of epigenetic research, affecting a wide range of physiological and pathological processes.
• RNA modification plays an important role in tumor immunity.
• RNA modification may be a potential clinical therapeutic target to prevent tumor immune escape.

## Graphical abstract

**Figure:**
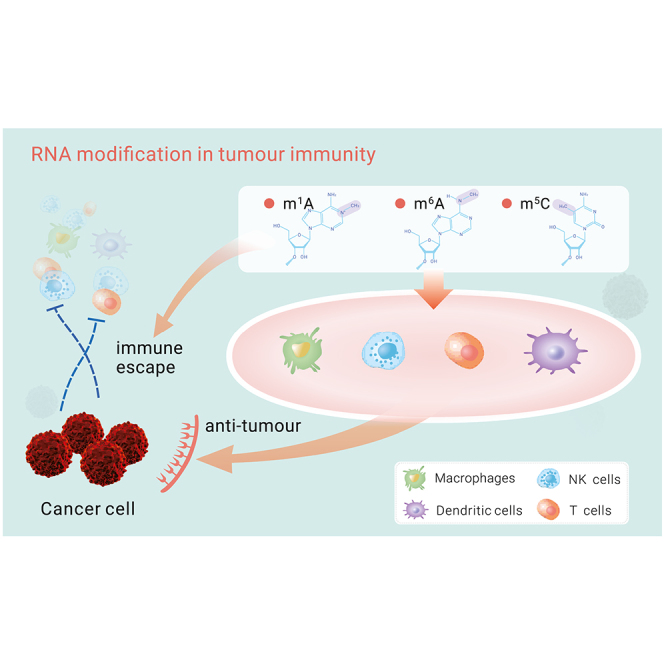

## Introduction

The immune system plays a crucial role in host defense against tumors. Antitumor responses mainly rely on activated CD8^+^ T cells recognizing tumor antigens. In addition, innate immune cells are pivotal in eliminating tumors. Despite the immune system aiming to eliminate tumor cells, tumor cells with reduced immunogenicity in an immunosuppressive tumor microenvironment (that is, one that includes immunosuppressive cells and immunosuppression-related molecules) can escape T cell attack. Immune checkpoint inhibitor (ICI) therapy can overcome immune escape and provide significant benefits to patients, and its application has revolutionized the field of tumor therapy. However, despite ICI effectiveness, most patients have a poor response to ICIs (primary resistance), and some patients who initially respond develop resistance after a period of treatment (secondary resistance), highlighting the necessity to explore novel treatment strategies to enhance the anticancer potency of immunotherapies.

RNA modification, a crucial epigenetic change, has become a research hotspot since its discovery in the 1950s.^1^ To date, more than 100 chemical modifications of RNA have been identified;^2^ these modifications include methylation to generate N1-methyladenosine (m^1^A),^3^ N6-methyladenosine (m^6^A),^4^ 5-methylcytosine (m^5^C),^5^ and N7-methylguanosine (m^7^G);^6^ RNA cap methylation;^7^ pseudouridine formation;^8^ and uridylation.^9^ The enzymes that catalyze the addition and removal of these modifications from RNA are called “writers” and “erasers,” respectively, and the RNA-binding proteins that identify modified RNA modifications are called “readers.” All types of RNA modifications are regulated by “readers,” “writers,” and “erasers.” These modifications play multifunctional roles in the RNA life cycle, affecting factors such as translation efficiency, transcript stabilization, pre-mRNA splicing, nuclear export, and mRNA storage under stress.^4^^,^^10^^,^^11^^,^^12^^,^^13^^,^^14^^,^^15^ Hence, RNA modifications affect a wide range of physiological and pathological processes.^13^^,^^16^^,^^17^

Notably, the combination of epigenetic drugs with immunotherapy may be a useful strategy to circumvent ICI resistance. For example, the combination of pembrolizumab (a PD-L1 inhibitor) plus entinostat (a histone deacetylase inhibitor) provides a clinically meaningful benefit to non-small cell lung cancer (NSCLC) patients who have shown resistance to PD-L1 inhibitors.^18^ In addition, administration of guadecitabine (a DNA methylation inhibitor) combined with pembrolizumab to patients with an advanced solid tumor can reverse previous resistance to ICIs.^19^ The positive results of these clinical trials indicate the importance of epigenetics in tumor immune regulation. Notably, RNA modifications have also been found to play a critical role in tumor immunity. For example, methyltransferase-like protein 3 (METTL3), a typical m^6^A reader, inhibits tumor growth and metastasis by enhancing the YTHDF1-mediated translation of SPRED2.^20^ Moreover, YTHDF1, a typical m^6^A reader, impairs tumor antigen cross-presentation of dendritic cells (DCs) by enhancing the translation of lysosomal cathepsin transcripts.^21^ Although numerous studies have been performed, details regarding the roles of RNA modifications in regulating tumor immunity remain unclear. Some reviews have summarized the critical roles of m^6^A in tumorigenesis, progression,^22^ and cancer metabolism.^23^ However, the roles of m^6^A and other RNA modifications (including m^1^A and m^5^C) in tumor immunity and the underlying mechanisms have not been systematically described, and we attempt to provide an up-to-date and comprehensive overview of these topics in this review.

In this review, we outline the evidence showing that m^6^A, m^1^A, and m^5^C modifications participate in tumor immunity by directly regulating different immune cells as well as by indirectly regulating tumor cells. Our review emphasizes the essential roles played by RNA modifications in shaping tumor immunity and highlights potential therapeutic strategies to prevent immune escape of tumor cells by targeting RNA modifications.

## The m^6^A modification in tumor immunity

### Overview of the m^6^A RNA modification

The m^6^A modification is the most prevalent RNA modification on mRNA and regulates the fate of mRNA by affecting its stability, splicing, translation, and export. It plays an important role in tumour immunity (Table 1). M^6^A was first discovered in 1974^24^ and is now considered the most representative type of RNA modification, accounting for 0.1%–0.4% of all adenosine molecules in total cellular RNA, with an average of 3–5 m^6^A modifications per mRNA.^25^ RRACH (R = G or A; H = A, C, or U) is the consensus sequence in mRNA on which the m^6^A modification is found, and a combination of immunoprecipitation and high-throughput sequencing results has revealed that the m^6^A modification is enriched at stop codons and 3′ UTRs.^26^ However, the consensus sequence for the m^6^A modification is different on noncoding RNAs (ncRNAs), including long ncRNAs (lncRNAs), microRNAs (miRNAs), circular RNAs (circRNAs), small nuclear RNAs (snRNAs), and ribosomal RNAs (rRNAs).^27^^,^^28^ Based on the known m^6^A modification sites, several RNA modification databases have been established (Table 2), and various m^6^A modification site prediction algorithms have been proposed (Table 3), such as WHISTLE,^29^ SRAMP,^30^ BERMP,^31^ iRNA-PseColl,^32^ and Gene2vec.^33^ Despite these advances, the specificities of m^6^A modification sites are still poorly understood.

**Table 1:** Functions of m^6^A regulators in tumor immunity

	Regulators	Cancer type	Function	References
Writers	METTL3	breast cancer	enhance PD-L1 expression	Wan et al.^104^
		bladder cancer	enhance PD-L1 expression	Ni et al.^105^
		NSCLC	reduce degradation of PD-L1	Liu et al.^106^
		colorectal carcinoma	reduce recruitment of CD8^+^ T cells and the levels of IFN-γ, CXCL9, and CXCL10	Wang et al.^121^
		colorectal cancer	facilitate MDSC accumulation by promoting CXCL1 expression	Chen et al.^122^
	RBM-15	ccRCC	increase macrophage infiltration and M2 polarization by promoting CXCL11 expression	Zeng et al.^123^
	METTL14	CCA	reduce degradation of PD-L1	Zheng et al.^107^
		HCC	enhance PD-L1 expression	Peng et al.^108^
		colorectal carcinoma	reduce recruitment of CD8^+^ T cells and the levels of IFN-γ, CXCL9, and CXCL10	Wang et al.^121^
Readers	IGF2BP1	HCC	enhance PD-L1 expression	Liu et al.^109^
	YTHDF1	colon cancer	enhance PD-L1 expression	Li et al.^110^
		GC	reduce DC recruitment and T cell infiltration by reducing expression of IFNGR1	Bai et al.^115^
	YTHDF3		downregulate PD-L1 expression	Zhao et al.^113^
Erasers	ALKBH5	ICC	enhance PD-L1 expression	Qiu et al.^111^
		melanoma/colon cancer	reduce sensitivity to immunotherapy	Li et al.^118^
		GBM	promote secretion of CXCL8/IL8 and recruitment of TAMs	Dong et al.^124^
		HCC	increase PD-L1^+^ macrophage recruitment	You et al.^125^
			Promote tumor progression by reducing RIG-I expression	Jin et al.^126^
	FTO	melanoma	enhance PD-1 (PDCD1), CXCR4, and SOX10 expression	Yang et al.^112^
		leukemia	maintain immune evasion by enhancing LILRB4 expression	Su et al.^114^
		melanoma	suppress infiltration and function of CD8^+^ T cells	Liu et al.^119^

**Table 2:** RNA modification databases

Database	Resources	Website	References
m6A2Target	m^6^A modifies the associated writers/erasers/readers	http://m6a2target.canceromics.org/#/	Bao et al.^220^
m6A-Atlas	m^6^A sites and quantitative epitranscriptome profiles	http://180.208.58.66/m6A-Atlas/index.html	Tang et al.^221^
REPIC	m^6^A-seq and MeRIP-seq data	https://repicmod.uchicago.edu/repic	Liu et al.^222^
DirectRMDB	Oxford Nanopore Technologies (ONT)-based database of quantitative RNA modification profiles	http://www.rnamd.org/directRMDB/	Zhang et al.^223^
MODOMICS	Modified residues, enzymes, guide RNAs, RNA modification pathways, sequences of modified RNAs, diseases	https://iimcb.genesilico.pl/modomics/	Boccaletto et al.^202^
RMVar	Multiple modification types, lncRNA/circRNA/miRNA methylation-associated SNPs/SNVs, RNA bing proteins (RBPs), diseases	http://rmvar.renlab.org/	Luo et al.^224^
RMDisease V2.0	1,366,252 RM-associated variants that may affect 16 different types of RNA modifications, diseases	www.rnamd.org/rmdisease2	Song et al.^225^
RMBase	Multiple methylation types, SNP/SNVs, RBPs, miRNA modifications	http://rna.sysu.edu.cn/rmbase/	Xuan et al.^226^
m^5^C-Atlas	m^5^C sites, associated SNPs, relevance to RNA secondary structure, RBPs	https://www.xjtlu.edu.cn/biologicalsciences/m5c-atlas	Ma et al.^227^

**Table 3:** RNA modification site prediction methods

Tool	Modification	Algorithm	Website	References
WHISTLE	m^6^A	SVM	www.xjtlu.edu.cn/biologicalsciences/whistle; http://whistle-epitranscriptome.com	Chen et al.^29^
SRAMP	m^6^A	RF	http://www.cuilab.cn/sramp/	Zhou et al.^30^
BERMP	m^6^A	BGRU	http://www.bioinfogo.org/bermp	Huang et al.^31^
iRNA-PseColl	m^6^A	SVM	http://lin-group.cn/server/iRNA-PseColl/	Feng et al.^32^
Gene2vec	m^6^A	CNN	http://server.malab.cn/Gene2vec/	Zou et al.^33^
iRNA-3typeA	m^1^A m^6^A	SVM	http://lin-group.cn/server/iRNA-3typeA/	Chen et al.^228^
RAMPred	m^1^A	SVM	http://lin-group.cn/server/RAMPred	Chen et al.^134^
M1ARegpred	m^1^A	SVM	https://github.com/SXWuFJMU/m1ARegpred.	Yao et al.^133^
iRNA-m5C	m^5^C	RF	http://lin-group.cn/server/iRNA-m5C/service.html	Lv et al.^229^
RNAm5Cfinder	m^5^C	RF	http://www.rnanut.net/rnam5cfinder	Li et al.^230^
M5C-HPCR	m^5^C	HPCR	http://cslab.just.edu.cn:8080/M5C-HPCR/	Zhang et al.^231^
m6AmPred	m^6^Am	XgbDart	https://www.xjtlu.edu.cn/biologicalsciences/m6am	Jiang et al.^232^
MultiRM	Multiple types	OHEM	www.xjtlu.edu.cn/biologicalsciences/multirm	Song et al.^233^

**Figure 1:**
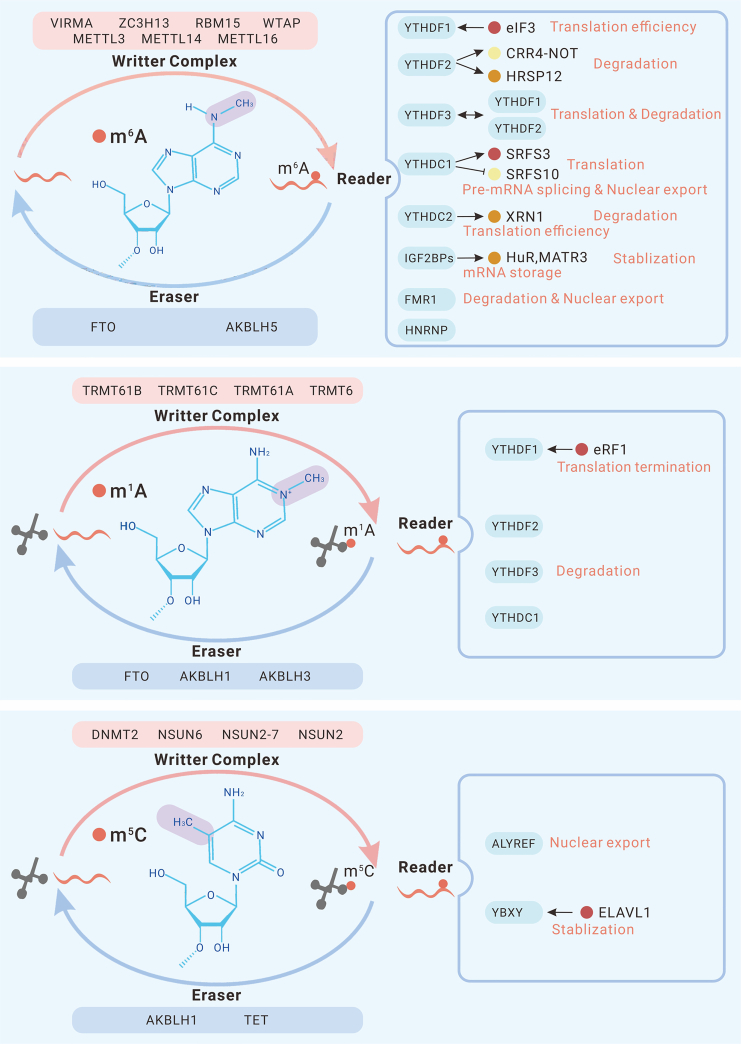
Regulators of RNA modifications and their molecular functions All RNA modifications are catalyzed by writers and removed by erasers. They can be recognized by their respective reader proteins and affect the fate of target RNAs by regulating their nuclear export, splicing, translation, and degradation.

To understand the roles of RNA modifications, appropriate analytical methods or techniques are essential. The lack of specific antibodies limits functional exploration to some extent. However, considerable improvements in methods for deciphering RNA modifications have been achieved in the last decade.^34^^,^^35^^,^^36^ The methods for detection of RNA modifications have evolved from liquid chromatography,^37^ reverse-transcriptase polymerase chain reaction (RT-PCR),^38^ and thin-layer chromatography (TLC)^39^ to mass spectrometry (MS)^40^ and sequencing analysis.^41^ Liquid chromatography (LC) coupled to MS (LC-MS) or tandem MS (LC-MS/MS) are the most commonly used MS methods to quantify RNA modifications on single nucleosides.^36^ Recently, an LC-electrospray ionization (ESI)-MS/MS method was developed. It was used to profile and characterize the RNA modifications in plant 18S rRNA and 25S rRNA.^42^ In addition, this method was also used to detect modifications of RNA in rat peripheral blood during alcohol exposure^43^ and in thyroid carcinoma tissue.^44^

m^6^A modifications are reversible and regulated by three types of enzymes: m^6^A methyltransferases (“writers”), m^6^A demethylases (“erasers”), and m^6^A-binding proteins (“readers”). The deposition of a methyl group to form m^6^A relies on the m^6^A methyltransferase complex (MTC), comprising the METTL3-methyltransferase-like protein 14 (METTL14) heterodimer and other adaptor proteins, including WT1-associated protein (WTAP), RNA-binding motif protein 15 (RBM15), vir-like m^6^A methyltransferase associated (VIRMA) subunits, and zinc-finger CCCH domain-containing protein 13 (ZC3H13).^45^^,^^46^ METTL3, discovered in 1999, was the first identified m^6^A methyltransferase.^47^ An S-adenosyl methionine (SAM)-dependent methyltransferase catalyzes RNA methylation using SAM, a prevalent methyl donor *in vivo*, and is critical for almost all m^6^A modifications on mRNA. In 2014, METTLE14 and WTAP were identified as other important MTC subunits.^48^^,^^49^ In the complex, METTL3 is the catalytic core, METTL14 is the RNA-binding platform, and WTAP is crucial for recruitment of the MTC.^45^^,^^49^ In addition to the aforementioned complexes and factors, the functions of methyltransferase-like protein 16 (METTL16) and the METTL5-TRMT112 complex have also been validated.^50^ m^6^A demethylases can remove a methyl group from m^6^A modifications on mRNA and cooperate with m^6^A methyltransferases to maintain the dynamic balance of m^6^A modifications. Fat mass and obesity-associated protein (FTO) and alkB homolog 5 (ALKBH5) are two well-known m^6^A demethylases.^51^^,^^52^ In general, m^6^A modifications are involved in functions via their recognition by m^6^A-binding proteins, including five members of the YTH domain-containing family (YTHDF1, YTHDF2, YTHDF3, YTHDC1, and YTHDC2),^53^ insulin-like growth factor 2 mRNA-binding proteins (IGF2BP1, IGF2BP2, and IGF2BP3),^15^ heterogeneous nuclear ribonucleoprotein proteins (HNRNPA2B1 and HNRNPC),^54^^,^^55^ leucine-rich pentatricopeptide repeat-containing (LRPPRC),^56^ fragile X mental retardation 1 (FMR1),^57^ and proline-rich coiled-coil 2 C (Prrc2c).^58^ Their roles are described in detail below.

m^6^A modifications affect the fate of mRNAs by regulating and controlling gene expression in various ways in different subcellular locations, as facilitated by reader proteins. (Figure 1) YTH domain-containing family members are the principal readers. The YTH structural domain recognizes and binds m^6^A modification sites. YTHDF1 interacts with translation initiation factor complex 3 (eIF3) and enhances mRNA translation efficiency,^4^ whereas YTHDF2 accelerates target transcript degradation in two ways: deadenylation mediated by carbon catabolite repression 4 (CCR4)-negative on TATA-less (NOT)^10^ complex action and endoribonucleolytic cleavage mediated by heat-responsive protein 12 (HRSP12).^11^ YTHDF3 cooperates with YTHDF1 to enhance protein synthesis by interacting with ribosomal proteins and increases YTHDF2-mediated mRNA decay,^59^ suggesting that YTHDF proteins interact with each other to synthetically influence fundamental biological processes associated with m^6^A RNA modification.^60^ Moreover, multiple m^6^A-modified mRNAs form phase-separated YTHDF-m^6^A-mRNA complexes by binding to YTHDF1–YTHDF3, which, in turn, form phase-separated structures in cells and may function during cellular stress to induce storage of certain mRNAs encoding cellular repair proteins.^61^ YTHDC1 facilitates exon inclusion in targeted mRNAs by recruiting arginine/serine-splicing factor 3 (SRFS3) and blocking serine/arginine-splicing factor 10 (SRSF10) binding, thus promoting mRNA translation.^62^ In addition, YTHDC1 affects mRNA density by splicing pre-mRNA transcripts^13^ and mediating the export of mRNA.^14^ YTHDC2 also enhances the translation efficiency of target mRNAs, shows 3'→5′ RNA helicase activity, and recruits XRN1, which is a 5'→3′ exoribonuclease, by inserting ankyrin repeats.^63^ In contrast to the decay-promoting function of YTHDF2, IGF2BPs (IGF2BP1–IGF2BP3) have been shown to stabilize target mRNAs in a manner facilitated by their cofactors (HuR and MATR3) and to promote the storage of target mRNAs under stress.^15^ In a previous study, FMR1 was found to bind to m^6^A to enhance mRNA stability.^12^ However, a recent study revealed that *Drosophila* FMR1 bound preferentially to mRNAs containing the m^6^A-tagged “AGACU” motif, forming FMR1-ribonucleoprotein (RNP) granules that then underwent a dynamic phase switch to promote mRNA decay.^64^ FMR1 has also been proven to promote nuclear export of m^6^A-modified mRNAs.^65^ As mentioned above, m^6^A-binding proteins play different or even contradictory roles in mRNA modification. YTHDF2 promotes mRNA degradation, but IGF2BPs have the opposite effect. In addition, in older and more recent studies, FMR1 has been reported to exert the opposite effect on mRNA stability. In addition, interactions between different RNA-binding proteins can be either cooperative or competitive.^57^^,^^59^ These complex regulatory roles may explain the diversity of m^6^A mechanisms of action and biological effects.

In addition to influencing the fate of mature mRNAs, m^6^A modifications regulate the generation and function of ncRNAs, which are also of physiological importance. Among miRNAs, HNRNPA2B1 is involved in regulating variable shearing of exons and subsequent processing of certain primary miRNAs (pri-miRNAs) by binding to the microprocessor complex DGCR8.^54^ Some lncRNAs are closely related to cancers and are regulated by RNA-modification-related enzymes, such as DMDRMR and MALAT1.^66^ Moreover, recent studies on m^6^A have focused on its interaction with the nucleus and chromatin as well as its role in nascent RNA processes, by which it can regulate gene transcription. m^6^A modifications on the lncRNA X-inactive-specific transcript (XIST) placed by RBM15 participate in transcriptional repression of X chromosome gene expression.^67^ m^6^A modifications on chromosome-associated regulatory RNAs (carRNAs) deposited by METTL3 and YTHDC1 accelerate the decay of these RNAs.^68^ This process is involved in regulating the state of nearby chromatin and downstream transcription.^68^ Regarding enhancer RNAs (eRNAs) and nascent transcripts, m^6^A-modified eRNAs can recruit the nuclear m^6^A reader YTHDC1 and promote formation of BRD4, which is related to enhancer and gene activation,^69^ and the m^6^A modification of nascent transcripts protects them from termination mediated by the integrator complex, thus enhancing gene expression.^70^ Moreover, m^6^A can bind to YTHDC1, and YTHDC1 can recruit KDM3B via physical interaction, which promotes histone dimethylated histone H3 Lys9 (H3K9me2) demethylation and gene expression.^71^

Overall, m^6^A modification of mRNAs and ncRNAs is involved in regulation of almost all important life processes. Recently, m^6^A modifications have been shown to regulate tumor immunity, and details regarding the regulatory network housing tumor immune factors and m^6^A modifications are described below. Collectively, m^6^A modifications regulate the immune response through two main processes: by directly affecting the function of immune cells and by indirectly regulating tumor cells. In the following sections, both of these mechanisms are outlined.

### m^6^A regulates tumor immunity by directly affecting immune cells

#### Macrophages

**Figure 2:**
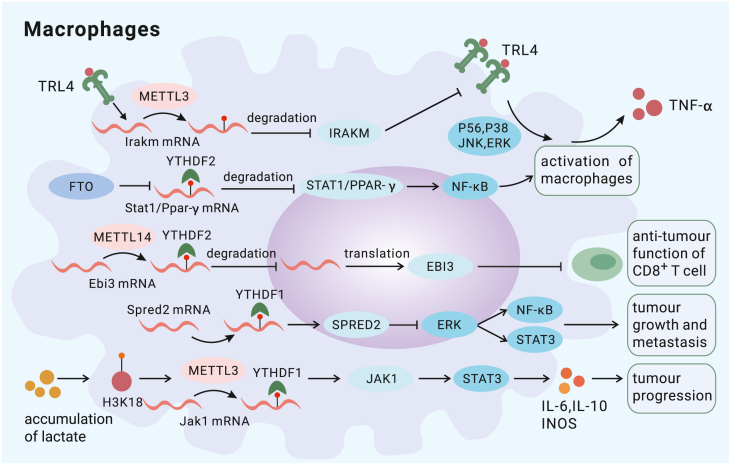
The mechanisms by which m^6^A modification regulates macrophages METTL3 (a writer) promotes production of TNF-α by enhancing degradation of Irakm (an inhibitor of TRL4) mRNA, and it also inhibits tumor growth by targeting SPRED2 (an inhibitor of the ERK pathway). However, METTL3 enhances immunosuppression through the Jak1/STAT3 pathway. METTL14 (a writer) increases degradation of Ebi3 mRNA, promoting the antitumor function of CD8^+^ T cells. FTO (an eraser) reduces degradation of STAT1/PPAR-γ mRNA, promoting macrophage activation.

Macrophages, vital components of innate immunity, can be polarized into different phenotypes depending on their genetic background and stimuli in the environment. A general classification of macrophages places these cells into the classically activated macrophage (M1) and alternatively activated macrophage (M2) categories.^72^ M1 macrophages show high antigen-presenting and T cell activation abilities, and M2 macrophages are related to immunosuppression.^73^ Recent studies have revealed that m^6^A is involved in regulating polarization of macrophages under physiological and inflammatory conditions. METTL3 drives M1 polarization by enhancing the stability of signal transducer and activator of transcription 1 (STAT1) mRNA.^74^ However, IGF2BP2 facilitates the switch of M1 macrophages into M2 macrophages by stabilizing tuberous sclerosis complex 1 (TSC1) and peroxisome proliferator-activated receptor γ (PPARγ).^75^ In addition to affecting polarization, m^6^A regulates the activation of macrophages. METTL3 has been identified as a positive regulator. For example, deletion of METTL3 in macrophages impairs their ability to eliminate pathogens because of reduced degradation of *Irakm* transcripts and subsequent inhibition of the Toll-like receptor 4 (TLR4) signaling pathway.^76^ However, FTO, a demethylase, activates macrophages by enhancing the stability of STAT1 and PPARγ in a YHTDF2-dependent manner.^77^ Moreover, m^6^A inhibits activation of certain cellular programs, with METTL14 and FTO preventing their hyperactivation. METTL14 mediates m^6^A methylation of suppressor of cytokine signaling 1 (SOCS1) mRNA and promotes translation of SOCS1 by binding to YTHDF1, an interaction that is essential for sustaining the negative feedback loop established via macrophage activation.^78^ In addition, knocking out FTO in macrophages mediates similar inflammation inhibition and leads to increased m^6^A abundance on SOCS1 mRNA, thereby facilitating transcript stability and subsequent translation in a YHTDF1-dependent manner^79^ (Figure 2).

Tumor-associated macrophages (TAMs) acquire an M2 phenotype and either infiltrate tumors or reside near tumor tissues. TAMs promote tumor progression and subvert antitumor immunity.^80^ Recently, the mechanisms by which m^6^A regulates the function of TAMs have been further elucidated. METTL3 and METTL14 are crucial for maintaining the antitumor function of macrophages. Deletion of METTL3 in myeloid cells creates an environment conducive to tumor growth and metastasis by increasing infiltration of immunosuppressive cells.^20^ Further research revealed that METTL3 deficiency in macrophages leads to a reduction in YTHDF1-mediated translation of SPRED2 and inhibition of the nuclear factor κB (NF-κB) pathway.^20^ Another study highlighted that C1q^+^ TAMs with high expression of METTL14 and YTHDF2 express diverse immunomodulatory ligands, which enhances the recruitment and function of CD8^+^ effector T cells (TEFFs).^81^ Moreover, METTL14-deficient C1q^+^ TAMs show elevated Ebi3, a cytokine subunit that negatively regulates T cell function, which results in CD8^+^ T cell dysfunction.^81^ On the other hand, m^6^A readers, including IGF2BP2 and YTHDF1, have been shown to play a significant inhibitory role in macrophage antitumor ability. By binding to IGF2BP2, the lncRNA PACERR induces pro-oncogenic effects and promotes M2 polarization in pancreatic cancer by stabilizing KLF12 and c-myc in the cytoplasm.^82^ In addition, METTL3 enhances the immunosuppressive function of tumor-infiltrating myeloid cells (TIMs) through the Jak1/STAT3 pathway by promoting Jak1 translation through the m^6^A-YTHDF1 axis.^83^ This process may be driven by lactate accumulation in the form of H3K18 lactylation marks^83^ (Figure 2).

#### DCs

**Figure 3:**
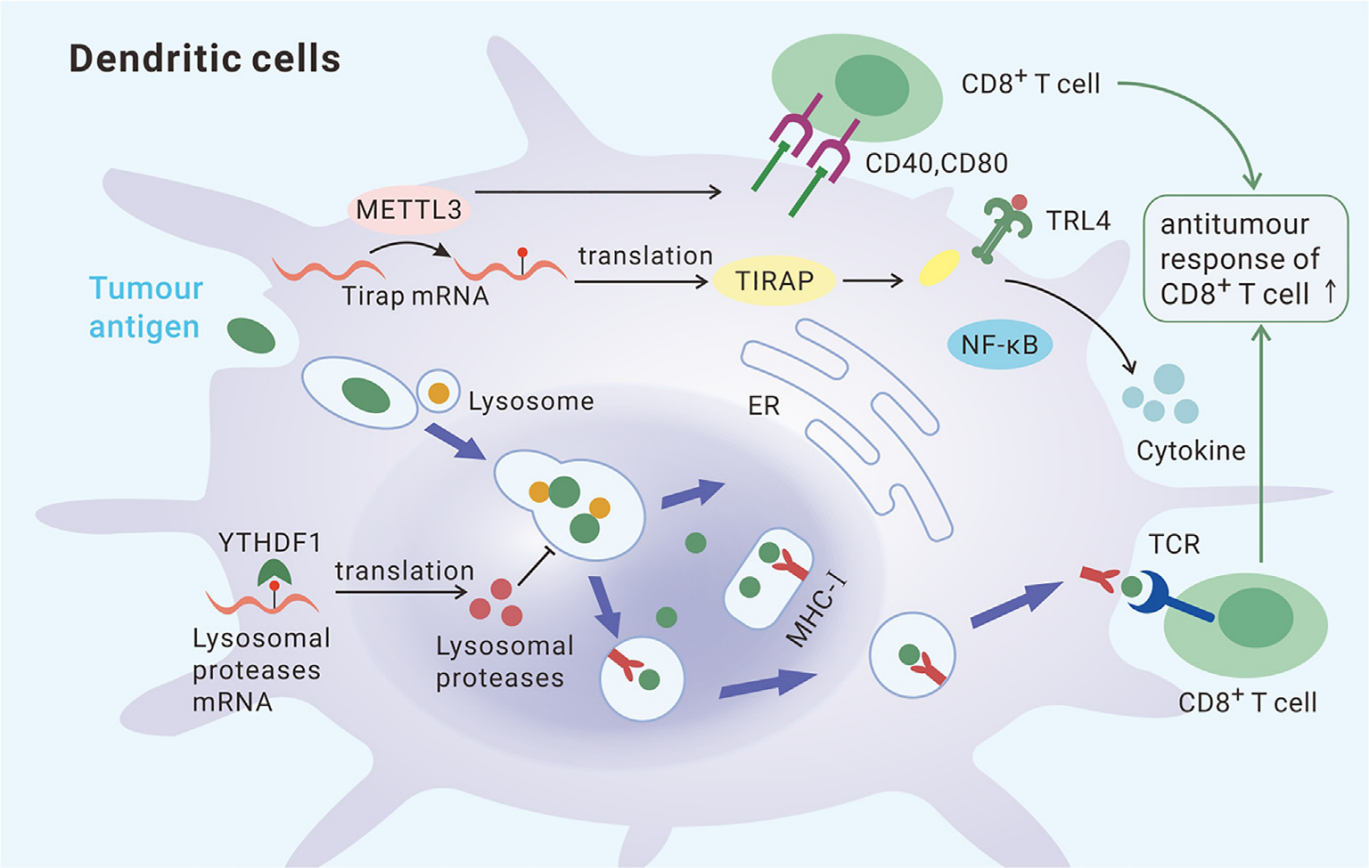
The mechanisms by which m^6^A modification regulates DCs METTL3 positively regulates the function of dendritic cells (DCs) by increasing CD40, CD80, and Tirap (TLR4 signaling adaptor) translation. YTHDF1 inhibits the cross-priming ability of DCs targeting lysosomal proteases, suppressing the CD8^+^ T cell antitumor response.

DCs are antigen-presenting cells (APCs) that process antigenic information, thereby connecting innate and adaptive immune responses.^84^ Recent studies have provided some clues about how m^6^A regulates stimulation, migration, and presentation of DCs under physiological conditions. METTL3-mediated mRNA m^6^A methylation enhances DC-mediated stimulation of T cell activation and cytokine production by enhancing the translation efficiency of CD40, CD80, and TLR4 signaling adaptor (Tirap) mRNA.^85^ In addition to affecting stimulation, m^6^A regulates DC migration. CC-chemokine receptor 7 (CCR7) is critical for DC migration to draining lymph nodes,^86^ and a feedback mechanism that inhibits CCR7-mediated DC migration in a manner mediated via m^6^A modification has been identified.^87^ Demethylation of the lncRNA Dpf3 induced by CCR reduces Dpf3 decay. Dpf3 binds to hypoxia-inducible factor 1 alpha (HIF-1α), impairing the glycolytic metabolism and migratory capacity of DCs by attenuating expression of the glycolytic gene *Ldha*^87^ (Figure 3).

Other studies have focused on the tumor background. For example, on the basis of positive regulation of DC function by m^6^A, researchers have designed nanomedicines containing an FTO inhibitor, and such agents promote DC maturation and antigen presentation after thermal ablation of hepatocellular carcinoma (HCC) cells.^88^ However, contrary to previous findings, YTHDF1 has been found to be a negative regulator of DC antitumor effects. YTHDF1-silenced mice show improved tumor antigen cross-presentation by DCs and antitumor responses by CD8^+^ T cells.^21^ Mechanistically, YTHDF1 enhances translation of lysosomal cathepsin transcripts, which contributes to antigen degradation, thereby reducing DC cross-priming capacity^21^ (Figure 3).

#### Natural killer cells

**Figure 4:**
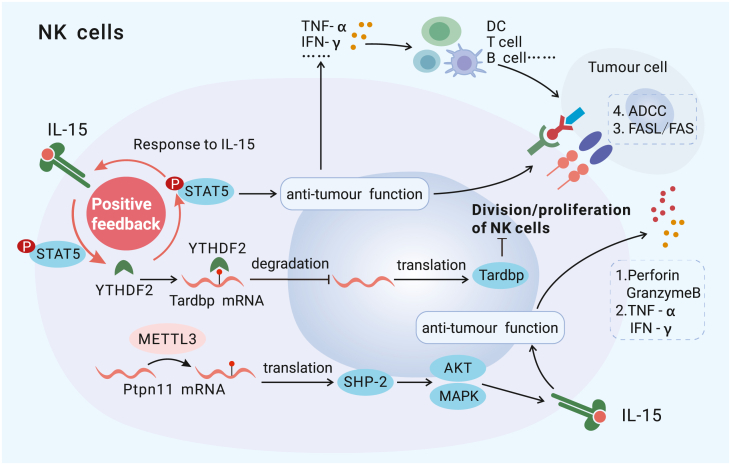
The mechanisms by which m^6^A modification regulates NK cells METTL3 enhances the antitumor function of NK cells by promoting SHP-2 (a critical mediator of IL-15-induced activation) translation. YTHDF2 (a writer) positively regulates NK cell division/proliferation by increasing Tardbp mRNA degradation. The STAT5–YTHDF2 positive feedback loop, which is downstream of IL-15, controls NK cell effector functions.

Natural killer (NK) cells constitute an important part of the innate immune system and are essential effector cells in antitumor immunity because they exert cytotoxic effects on tumor cells through surface receptors.^89^^,^^90^ They also regulate the antitumor immune response by expressing cytokines and chemokines that influence the recruitment and action of other immune cells, such as T cells and DCs.^91^^,^^92^ Recent studies have shown that regulators of m^6^A consistently activate NK cells. YTHDF2 deficiency profoundly impairs the antitumor and antiviral capacities of NK cells.^93^ YTHDF2 plays multifaceted roles in NK cells, including maintaining homeostasis, regulating terminal maturation, and favoring response to interleukin-15 (IL-15) by enhancing the stability of *Tardbp* mRNA.^93^ In addition, METTL3 is an important positive regulator of NK cell antitumor effects because it maintains homeostasis and infiltration of NK cells.^94^ Conditional ablation of METTL3 in NK cells weakened the immune response to IL-15 exposure, and this mitigated response was related to reduced expression of Src-homology phosphotyrosine phosphatase-2 (SHP-2) mediated by a reduction in m^6^A abundance on *Ptpn11* mRNA^94^ (Figure 4).

#### T cells

**Figure 5:**
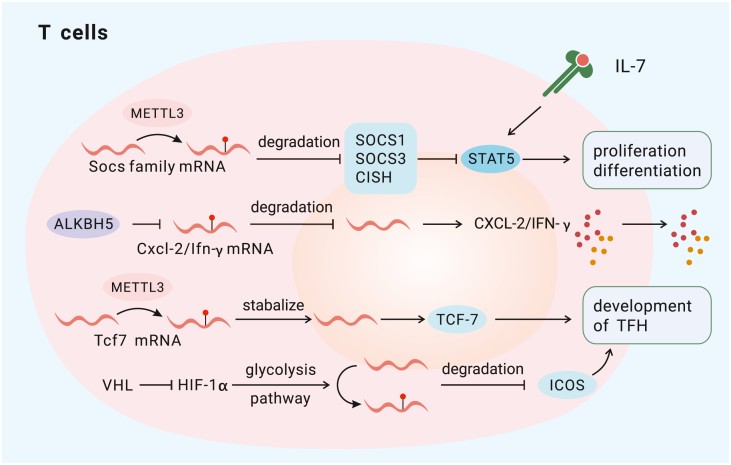
The mechanisms by which m^6^A modification regulates T cells METTL3 promotes proliferation and differentiation of T cells via the JAK/STAT5 pathway by decreasing the expression of SOCS family proteins (SOCS1, SOCS3, and CISH). ALKBH5 (an eraser) enhances production of CXCL-2 and IFN-γ by targeting CXCL-2 and IFN-γ mRNA degradation. METTL3 also promotes T follicular helper (Tfh) development by stabilizing Tcf-7 mRNA to increase TCF-1 (a regulator of Tfh differentiation) expression. The VHL-HIF-1α axis regulates Tfh cell development in an m^6^A-dependent manner.

**Figure 6:**
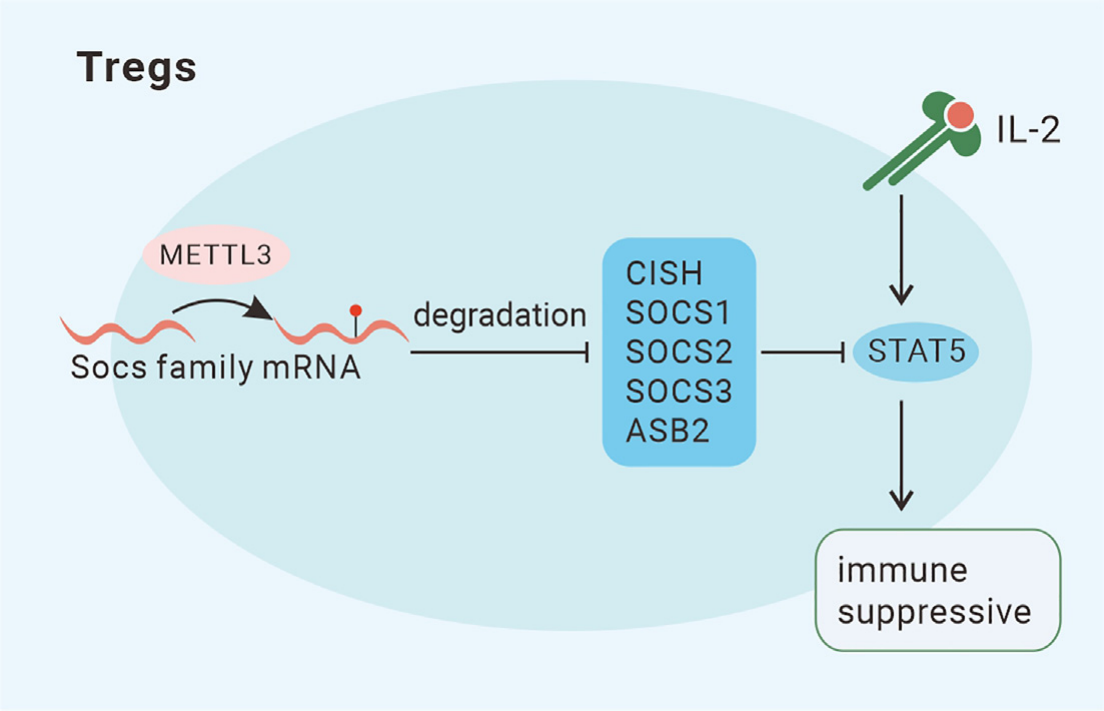
The mechanisms by which m^6^A modification regulates T cells METTL3 mediates immune suppression via the JAK/STAT5 pathway by decreasing the expression of SOCS family proteins (SOCS1, SOCS2, SOCS3, CISH, and Asb2).

T cells, including CD4^+^ T cells and CD8^+^ T cells, play essential roles in adaptive immunity. Regulatory T cells (Tregs) constitute a special T cell subpopulation that controls self-tolerance and is associated with immunosuppression.^95^ Recent studies have provided some clues to explain the association between m^6^A regulators and the differentiation, homeostasis, survival, and pathogenicity of T cells. First, deleting METTL3 in T cells disrupts CD4^+^ T cell homeostasis and differentiation by upregulating the expression of SOCS family members (Socs1, Socs3, and Cish), which encode proteins that inhibit STAT signaling and thus downregulate IL-7-mediated STAT5 activation^96^ (Figure 5). Similarly, in Tregs, METTL3 has been shown to contribute to immune suppression through the IL-2/STAT5 signaling pathway by regulating the expression of SOCS family members (Cish, Socs1, Socs2, Socs3, and Asb2)^97^ (Figure 6). Second, after targeted knockdown of WTAP, cell death is induced by activation of the T cell receptor (TCR) signaling pathway.^98^ WTAP knockdown stabilizes Orai1 and Ripk1 mRNAs, and subsequent cell death is mediated by more intense and sustained Ca^2+^ signaling.^98^ Third, ALKBH5 controls the pathogenicity of CD4^+^ T cells.^99^ Ablation of ALKBH5 in naive CD4^+^ T cells suppresses development of colitis and autoimmune diseases by reducing interferon-γ (IFN-γ) and CXCL2 mRNA stability^99^ (Figure 5).

T follicular helper (Tfh) cells are another specific subpopulation of CD4^+^ T cells; they initiate formation of germinal centers and promote secretion of high-affinity antibodies by B cells.^100^ Knocking out METTLE3 in CD4^+^ T cells leads to poor differentiation of Tfh cells and, thus, impaired germinal center responses.^101^ Further experiments elucidated that m^6^A modification of the 3′ UTR of Tcf7 mRNA is mediated by METTL3 and that this modification stabilizes Tcf7 transcripts and subsequently ensures activation of Tfh cells.^101^ However, in another study, m^6^A modifications have been shown to exert the opposite effects on Tfh cell development. Specifically, m^6^A modification induced by glyceraldehyde-3-phosphate dehydrogenase (GAPDH) accelerates degeneration of inducible costimulator (ICOS) mRNA and, thus, impairs Tfh cell initiation when von Hippel-Lindau (VHL) activity is impaired^102^ (Figure 5).

In addition to its effect on CD4αβ T cells, m^6^A regulates the development of γδ T cells. γδ T cells are involved in intrinsic immunity and are abundant in the mucosae, where they function in immune surveillance. m^6^A has been identified as a developmental checkpoint for immature γδ T cell development, and the number of γδ T cells has been shown to increase significantly when ALKBH5 is absent.^103^ Moreover, ALKBH5-mediated m^6^A demethylation decreases the stability and expression of Jagged1 and Notch2 mRNAs, inactivating the Jagged1/Notch2 signaling pathway.^103^

Above all, different m^6^A modification regulators play a variety of roles in different cells. For example, METTL3, the most classic m^6^A writer, has the most extensively studied function in immune cells, and its knockdown in macrophages, DCs, NK cells, and T cells impairs their functions. However, other m^6^A regulators, such as YTHDF2 in DCs, negatively regulate antitumor functions; therefore, inhibitors of these m^6^A regulators may be effective antitumor agents. Despite the diversity of roles of m^6^A modifications, there are common mechanisms regulating functions across different immune cells. First, m^6^A, as the most abundant modification on mRNA, regulates gene expression by regulating the stability, splicing, translation, and export of mRNAs. In immune cells, m^6^A mainly influences the translation efficiency and stability of mRNAs of related genes. Second, although the regulatory pathways are different, m^6^A modifications generally influence similar cellular processes in different immune cells, such as cell proliferation, differentiation, and secretion of cytokines, which ultimately affects their antitumor capacity.

In addition, interactions and synergies between different immune cells are worthy of attention. For example, CD8^+^ T cells, as the main antitumor effector cells, are downstream of many immune cell functions. Therefore, regulation of macrophage and DC function by m^6^A ultimately affects their antitumor function by influencing the recruitment, function, and activation of CD8^+^ T cells.^21^^,^^81^^,^^85^

### m^6^A regulates the immune response by affecting tumor cells

In addition to its direct regulatory role in a variety of immune cells, m^6^A regulates antitumor immunity by indirectly regulating tumor cell function. As recent studies have shown, three main mechanisms, surface molecule expression, cytokine secretion, and metabolism reprogramming, are involved in m^6^A regulation of tumor cells, and these mechanisms are described below.

### Expression of surface molecules

Tumor cells express surface molecules associated with tumor immunity, especially immune checkpoints such as PD-L1, CTLA-4, and CD276, which play important roles in regulating antitumor immunity by costimulating or coinhibiting the killing effect of T cells and are thus key targets for immunotherapy.

**Figure 7:**
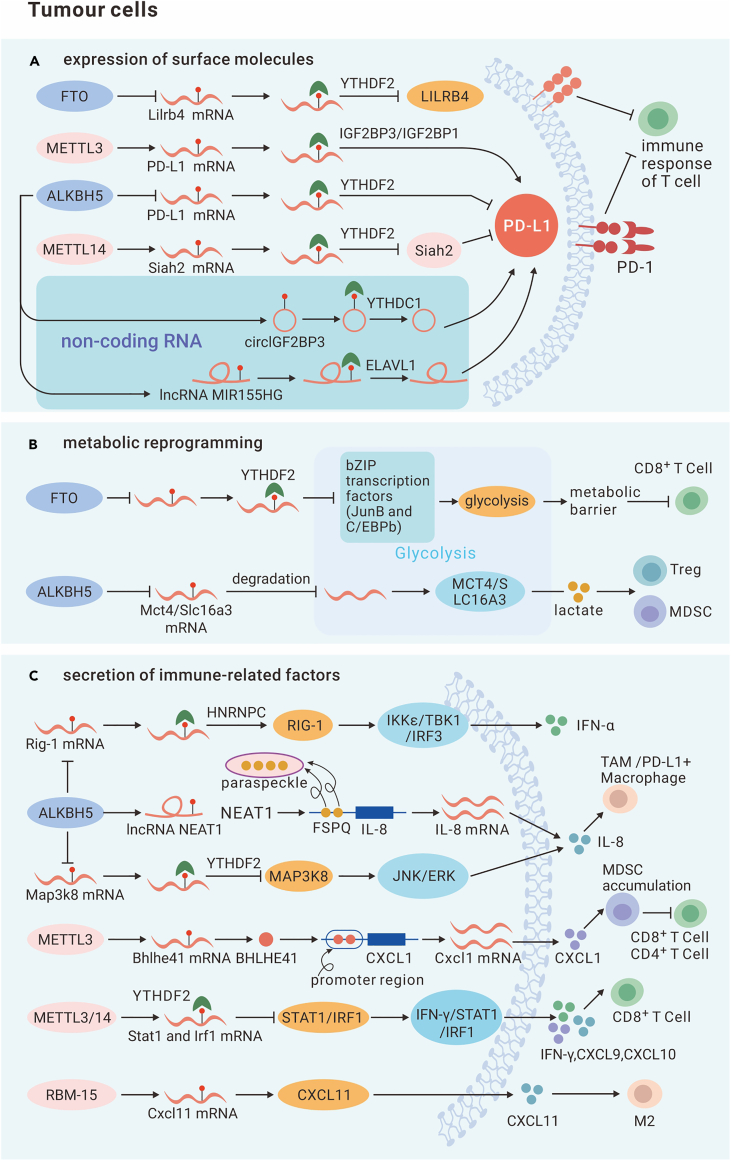
The mechanisms by which m^6^A modification induces immune evasion in tumor cells (A) FTO promotes ILIRB4 (an immune checkpoint) expression by targeting its mRNA. METTL3 and ALKBH5 upregulate the expression of PD-L1 by targeting PD-L1 mRNA, thus enhancing immunosuppression. METTL3 also regulates ncRNAs (circIGF2BP3 and lncMIR155HG). METTL14 negatively regulates Siah2 (a RING E3 ubiquitin ligase that enhances PD-L1 degradation) to increase PD-L1. (B) FTO increases the metabolic barrier for T cell activation and inhibits the function of CD8^+^ T cells targeting JunB and C/EBPb (glycolysis regulators). ALKBH5 enhances lactate content in tumor-infiltrating Tregs and MDSCs by targeting Mct4/Slc16a3. (C) ALKBH5 inhibits production of IFN-γ and IL-8 via the ALKBH5/RIG-I/IFNα axis, the ALKBH5/paraspeckle/CXCL8 axis, and the ALKBH5/MAP3K8 axis. METTL3 promotes CXCL1 production by targeting BHLHE4 (which binds to the promoter region of CXCL1) and induces immunosuppression. METTL3/14 also inhibits IFN-γ, CXCL9, and CXCL10 by inhibiting IFN-γ-STAT1-IRF1 signaling. RBM-15 promotes CXCL11 production, thus inducing M2 polarization.

Several studies have demonstrated that PD-L1 expression in tumor cells is closely correlated with m^6^A modification of RNA. METTL3, a typical m^6^A methyltransferase, suppresses tumor immune surveillance in breast cancer.^104^ Knocking out METTL3 results in decreased m^6^A modification abundance on PD-L1 mRNA and, thus, reduced PD-L1 mRNA stability, leading to less IGF2BP3 binding to PD-L1 mRNA and downregulating PD-L1 expression.^104^ Similarly, in bladder cancer, METTL3 induced c-Jun N-terminal kinase (JNK) signaling activation to stabilize PD-L1 mRNA by promoting the interaction between the m^6^A site and IGF2BP1.^105^ In addition, METTL3 also mediates immune escape by affecting ncRNA modifications. METTL3 mediates the m^6^A modification of circIGF2BP3 in NSCLC and promotes cyclization of circIGF2BP3 by binding to YTHDC1, thereby increasing the content of circIGF2BP3.^106^ This increase in the circIGF2BP3 level in NSCLC contributes to tumor cell immune evasion by increasing PD-L1 levels through reduced PD-L1 ubiquitination and subsequent proteasomal degradation.^106^ METTL14 is another important m^6^A writer. METTL14 reduces the degradation of PD-L1 via the ubiquitination-dependent pathway by destabilizing Siah2 mRNA in a YTHDF2-dependent manner in cholangiocarcinoma (CCA).^107^ In addition, in HCC, LPS-induced METLL14 expression increased m^6^A modification abundance on the lncRNA MIR155HG, thereby stabilizing MIR155HG by binding to ELAVL1 (HuR) and subsequently increasing PD-L1 expression via the miR-223/STAT1 axis.^108^ Regarding reader proteins, knockdown of IGF2BP1 reduced PD-L1 expression and promoted immune infiltration in HCC.^109^ In addition to the abovementioned regulators, methionine and YTHDF1 have been shown to promote PD-L1 expression.^110^ FTO and ALKBH5 are the two main m^6^A demethylases. Conditional deletion of the ALKBH5 gene in intrahepatic CCA (ICC) cells increases m^6^A abundance on PD-L1 mRNA and increases PD-L1 mRNA decay by binding to YTHDF2.^111^ Knocking down FTO in melanoma enhances sensitivity to anti-PD-1 and IFN therapies by suppressing PD-1 (PDCD1), CXCR4, and SOX10 expression through YTHDF2-mediated mRNA decay, but it does not affect PD-L1 (CD274).^112^ In general, METTL3, METTL14, IGF2BP1, FTO, and ALKBH increase the level of PD-L1 on the membrane and thereby promote tumor cell immune escape. In summary, these mechanisms of PD-L1 regulation can either directly upregulate PD-L1 expression or reduce PD-L1 protein degradation. Therefore, speculating that inhibitors of METTL3, IGF2BP, FTO, and ALKBH may be promising targets for cancer therapy is reasonable, and moreover, combination of these inhibitors with PD-1 inhibitors may enhance the efficacy of anti-PD-1 therapy to overcome drug resistance. This possibility has been confirmed by numerous studies.^109110^ However, m^6^A on CBX1 mRNA decreases its stability via YTHDF3 recognition, and CBX1 upregulates PD-L1 expression via the IFN-γ-STAT1 pathway,^113^ suggesting diverse functions of m6A in tumor immunity (Figure 7A; Table 1).

In addition to that of PD-1/PD-L1, the expression of other immune checkpoint ligands is regulated by m^6^A modifications. FTO has been shown to play a role in maintaining immune evasion in leukemia.^114^ LILRB4 is an important immune checkpoint, and reduced LILRB4 expression enhances T cell toxicity and antitumor immune responses. Inhibition or depletion of FTO increases degradation of m^6^A-modified LILRB4 mRNA, primarily by promoting m^6^A-modified LILRB4 mRNA binding to YTHDF2^114^ (Table 1).

In addition to the checkpoint ligands discussed above, expression of many other surface molecules regulates the immune response. YTHDF1 has been found to be overexpressed in gastric cancer (GC) and has been identified as an oncogene because of its induction of cell proliferation, and it has also been found to be associated with the antitumor immune response.^115^ Deletion of YTHDF1 in GC cells led to increased DC recruitment and T cell infiltration because of increased levels of IFNγ receptor 1 (IFNGR1) on the surface of tumor cells.^115^ However, it is not clear whether this effect is mediated by m^6^A.

### Metabolic reprogramming

Metabolic reprogramming of tumor cells can reshape the tumor microenvironment and thus affect the immune response; moreover, the vital role played by m^6^A in cancer metabolism has been fully proven.^116^^,^^117^ Notably, m^6^A controls tumor immunity via metabolic reprogramming, which is discussed below. Deletion of the m^6^A demethylase ALKBH5 has been found to increase the sensitivity of melanoma and colon cancer to immunotherapy.^118^ ALKBH5 deletion alters the metabolic profile of tumor cells and reduces lactate levels in the tumor immune microenvironment by altering the splicing and expression of target genes (Mct4/Slc16a3) via an m^6^A-dependent mechanism.^118^ This altered metabolism, in turn, leads to attenuation of recruitment of immunosuppressive cells such as Tregs and myeloid-derived suppressor cells (MDSCs).^118^ Another m^6^A demethylase, FTO, has also been found to be associated with tumor cell escape from immune surveillance through its effect on glycolytic metabolism.^119^ Notably, the FTO gene reduces the levels of basic leucine zipper (bZIP) transcription factors (JunB and CCAAT/enhancer binding protein-b(C/EBPb) ) in the m^6^A/YTHDF2 pathway in B16-OVA cells, which reduces gluconeogenic activity and facilitates the infiltration and function of CD8^+^ T cells by removing the metabolic barrier to their activation.^119^ YTHDF1 also potentiates glycolysis. circRHBDD1 is a novel circRNA that enhances the glycolysis rate and glycolysis capacity via the phosphatidylinositol 3-kinase (PI3K)/AKT pathway, and its inhibition enhances the anti-PD-L1 therapeutic effect.^120^ Mechanistically, circRHBDD1 promotes YTHDF1 binding to PIK3R1 mRNA, thereby facilitating PIK3R1 translation and activating downstream signaling pathways.^120^ At present, studies of how m^6^A modification affects metabolic reprogramming to alter immune responses are focused mainly on glucose metabolism, and the regulation of other tumor metabolism pathways needs to be investigated further (Figure 7B; Table 1).

### Secretion of immune-related factors and other mechanisms

The immune response can be regulated by immune-related factors, including chemokines, cytokines, and IFNs, in the tumor microenvironment. An increasing number of studies have proven the connection between m^6^A and these immune-related factors. For example, METTL3 or METTL14 deficiency in colorectal carcinoma cells increases recruitment of CD8^+^ T cells and the levels of IFN-γ, CXCL9, and CXCL10 in the tumor microenvironment.^121^ Mechanistically, STAT1 and INF1 mRNA stability is negatively regulated in an m^6^A-Ythdf2-dependent manner, and IFN-γ-STAT1-IRF1 signaling affect the immune response.^121^ In another study, METTLE3 facilitated MDSC accumulation in colorectal cancer by promoting CXCL1 expression and thereby compromised the function of CD4^+^ and CD8^+^ T cells.^122^ Notably, m^6^A regulates CXCL1 expression not by directly regulating CXCL1 mRNA but by promoting the translation efficiency of BHLHE41, a transcription factor that binds the promoter region of CXCL1.^122^ RBM-15 has been identified as an essential component of writer complexes. Similarly, RBM-15 has been shown to promote CXCL11 expression, although it accomplishes this through its direct regulation of CXCL11 mRNA stability, thereby increasing macrophage infiltration and M2 polarization.^123^ ALKBH5 has been identified as another regulator that fosters immunosuppression. It promotes secretion of CXCL8/IL-8 and recruitment of TAMs under anoxic conditions in a glioblastoma multiforme (GBM) model.^124^ ALKBH5 regulates CXCL8/IL-8 not by directly removing m^6^A modifications on CXCL8 mRNA but by stabilizing the lncRNA NEAT1; hence, the transcriptional repressor splicing factor proline and glutamine rich (SFPQ) is removed from the CXCL8 promoter and transferred to paraspeckles.^124^ Similarly, another study showed that ALKBH5 upregulates IL-8 expression in HCC, thereby increasing PD-L1^+^ macrophage recruitment and facilitating immunosuppressive microenvironment formation.^125^ Mechanistically, ALKBH5 destabilizes MAP3K8 mRNA in a YTHDF2-mediated manner and activates the JNK/extracellular signal-regulated kinase (ERK) pathway.^125^ In head and neck squamous cell carcinoma (HNSCC), knocking down ALKBH5 inhibits tumor progression. Depletion of ALKBH5 increases m^6^A abundance on DDX58 mRNA and increases retinoic acid-inducible gene I (RIG-I) expression by increasing DDX58 mRNA maturation in a manner dependent on the function of the m^6^A reader HNRNPC.^126^ RIG-I positively regulates secretion of IFNα through the inhibitor of kappa B kinase ε (IKKε)/TBK1/IRF3 pathway (Figure 7C; Table 1).

In addition, tumor cells regulate antigen expression and the IFN response through modulation of m^6^A modifications. The YY1-CDK9 transcription elongation complex affects the expression levels of METTL3 and YTHDF2, thus inhibiting the IFN response and antigen presentation in glioblastoma stem cells (GSCs).^127^ Mechanistically, IFN-related genes (IFNB1, STAT1, and IRF1) are stabilized in an m^6^A-dependent manner, and IFN signaling is enhanced when the complex is inhibited.^127^

In summary, the roles played by m^6^A regulators in tumor cells are more consistent than the complex roles they play in immune cells. As an immune evasion regulator in tumors, m^6^A upregulates the expression of immune checkpoints and promotes recruitment of immunosuppressive cells such as Tregs, MDSCs, and M2 macrophages, thus contributing to an immunosuppressive tumor microenvironment. Therefore, targeting the m^6^A pathway may be a new strategy for tumor immunotherapy.

## The m^1^A RNA modification in tumor immunity

### Overview of the m^1^A RNA modification

The m^1^A modification, which was first discovered decades ago, was initially recognized as a reliable way to regulate the function and stability of transfer RNA (tRNA) and rRNA.^128^^,^^129^^,^^130^ More recently, m^1^A modifications have been shown to mark eukaryotic mRNAs.^131^ However, in contrast to the m^6^A modification, which preferentially decorates stop codons and 3′ UTRs, the m^1^A modification is enriched mainly on start codons upstream of the first splice site and 5′ UTRs of mRNA transcripts.^131^^,^^132^ M1ARegpred^133^ and RAMPred^134^ are powerful tools for predicting m^1^A modification sites (Table 3).

Similar to the m^6^A modification, the m^1^A modification is also regulated by specific regulators. The methyl group in the m^1^A modification is mainly added to mitochondrial and cytoplasmic RNAs by specific writers. tRNA methyltransferase 6 noncatalytic subunit (TRMT6) and RNA methyltransferase 61A (TRMT61A) form the α2β2 heterotetramer TRMT6/61A,^135^ which is a methyltransferase targeting cytoplasmic tRNAs, while TRMT61B^136^ and TRMT10C^137^ are tRNA methyltransferases that act mainly in mitochondria.^138^ Moreover, TRMT6/TRMT61A and TRMT10C catalyze the m^1^A modification of mRNAs in the cytoplasm and mitochondria.^138^ In addition, nucleomethylin (NML) has been discovered to be a writer for 28S rRNA m^1^A modification.^139^ Some readers have also been identified. YTHDF proteins, first identified as m^6^A readers, have been shown to recognize and bind to the m^1^A modification.^140^ Their roles as m^1^A-binding proteins remain to be further elucidated. In terms of erasers, ALKBH1,^141^ ALKBH3,^142^ ALKBH7,^143^ and FTO^144^ can remove m^1^A on tRNA, while ALKBH3 can also target modifications on mRNA.^132^

Generally, m^1^A is crucial for the structure and function of tRNAs and mRNAs. m^1^A modifications were first identified on tRNAs, and the positive electrostatic charge carried by m^1^A has been shown to be essential for maintaining the structure and function of tRNAs.^145^ In a subsequent study, ALKBH1 was found to act as a demethylase that removes methyl groups from m^1^A modifications on tRNAs and tRNA^iMet^, thereby reducing translation initiation and translation elongation.^141^ Recently, m^1^A modifications were found on mRNAs and disrupted Watson-Crick base pairing.^131^ Their function on mRNAs is still being explored. Li et al.^132^ found that m^1^A on 5′ UTRs is associated with higher translational efficiency, whereas m^1^A on mitochondrial mRNAs, located mainly in the coding sequence (CDS), inhibits translation. Consistent with these findings, ALKBH3 knockdown results in the downregulation of ErbB2 and AKT1S1 expression.^146^ However, Safra et al.^138^ found that only low m^1^A abundance on mRNA in the cytosol results in translational repression. In addition to affecting the translation efficiency of the target mRNA, ALKBH3, a targeted mRNA m^1^A demethylase, reduces the stabilization of macrophage colony-stimulating factor (CSF-1) mRNA.^147^ RNA-binding proteins may play essential roles in the function of m^1^A. YTHDF1 binds to m^1^A on ATP5D mRNA and subsequently forms a complex with eRF1 to terminate translation.^148^ In addition, YTHDF2 and YTHDF3 have been demonstrated to reduce the stability of target transcripts^149^^,^^150^ (Figure 1).

### Aberrant m^1^A RNA modification in tumor immunity

Previous studies have revealed a relationship between m^1^A modification and tumorigenesis; for example, m^1^A modification of tRNAs drives liver tumorigenesis through activation of cholesterol synthesis.^151^ However, the relationship between m^1^A methylation and tumor immunity remains largely unknown. A recent study reported that tRNA-m^1^A modification was essential for rapid T cell proliferation.^152^ TRMT61A, which has been recognized as a typical m^1^A writer, is crucial for CD4^+^ T cell immune function, as indicated by an experiment in which its absence led to arrest of activated CD4^+^ T cells.^152^ Further studies revealed that this effect is mediated by tRNA-m^1^A enhancement of the translation efficiency of Myc mRNA, mainly mediated by an improved elongation process, which enhances expression of Myc and ultimately regulates cell cycling and metabolism.^152^ In addition, the analysis showed that m^1^A regulatory genes are positively correlated with immune cell infiltration and that YTHDF3, which had been identified previously as an m^1^A reader, may regulate macrophage activation.^153^ Bioinformatics studies have largely aimed to study m^1^A regulators, including three writers (TRMT6, TRMT61A, and TRMT10C), two erasers (ALKBH1 and ALKBH3), and four readers (YTHDF1, YTHDF2, YTHDF3, and YTHDC1). In oral squamous cell carcinoma,^154^ colon cancer,^155^ and HCC,^156^ the m^1^A score was found to be negatively correlated with patient prognosis and immune cell infiltration. In contrast, in ovarian cancer^157^ and lung adenocarcinoma,^158^ the m^1^A score was found to be positively correlated with prognosis and immunotherapy outcome. In ovarian cancer, innate immune cells were increased in the high m^1^A score subpopulation, whereas adaptive immune cells were increased in the low m^1^A score subpopulation.^157^ The m1A score showed a positive correlation with almost all immune cells in lung adenocarcinoma.^158^ The differences in these outcomes are rarely studied, and further research regarding recent theories is urgently needed.

## The m^5^C RNA modification in tumor immunity

### Introduction to the m^5^C RNA modification

The m^5^C RNA modification involves methylation of cytidine residues at position 5. It is widespread on rRNAs, tRNAs, other ncRNAs, and mRNAs. The m^5^C modification is conserved in tRNAs and rRNAs, but in mRNAs, the m^5^C modification is located primarily in 5′ UTRs and 3′ UTRs.^159^ Various m^5^C prediction models based on different machine learning algorithms have emerged; for example, Chen et al.^160^ predicted other modification sites with various machine learning models based on known m^5^C modification sites on mRNAs in humans and mice. Further predictive models are described in Table 3.

Similar to m^6^A and m^1^A modifications, m^5^C modifications are regulated by writers, readers, and erasers. m^5^C modification relies mainly on the NOL1/NOP2/sun (NSUN) family, which includes NSUN1,^161^ NSUN2,^162^ NSUN3,^163^ NSUN4,^164^ NSUN5, NSUN6,^165^ NSUN7, and the DNA methyltransferase homologue DNMT2.^166^ These writers mainly catalyze methylation on tRNAs (NSUN2, NSUN3, NSUN6, and DNMT2), rRNAs (NSUN1, NSUN4, and NSUN5), and other ncRNAs (NSUN2 and NSUN7) in the nucleus and mitochondria, while NSUN2^167^ and NSUN6^168^ mediate m^5^C modification on mRNAs. m^5^C function partially depends on RNA-binding proteins, and research in this area is still in an early stage. RNA and export factor-binding protein 2 (ALYREF), Y-box-binding protein 1 (YBX1), ypsilon schachtel (YPS), and YTHDF2 are the currently known m^5^C-binding proteins,^167^^,^^169^^,^^170^^,^^171^ and their functions are described below. Recently, ten-eleven translocation (TET) family members and ALKBH1 were identified as m^5^C demethylases that dynamically regulate m^5^C content.^172^

m^5^C controls the fate of modified RNA. Research on m^5^C initially focused on rRNAs and tRNAs. Regarding its effect on rRNAs, m^5^A regulates rRNA maturation,^171^ rRNA stability,^161^ and protein translation.^161^^,^^173^ In tRNAs, m^5^A is involved in stabilizing tRNA structure^174^ and regulating selective translation during oxidative stress.^175^ NSUN2 mediates methylation of tRNAs, reducing tRNA cleavage by nucleic acid endonucleases and thereby increasing tRNA stability, ultimately affecting translation efficiency.^176^ In addition, TET mediates oxidation of m^5^C in tRNAs and promotes translation.^177^ In recent years, with advances in detection methods, studies on m^5^C modification of mRNAs have gradually increased. NSUN2 has been found to also mediate m^5^C modification of mRNAs; ALYREF is an mRNA export adaptor that specifically binds to the m^5^C site after mRNA m^5^C modification to regulate targeted mRNA export.^167^ In addition, ALYREF enhances the stability of pyruvate kinase M2 (PKM2) mRNA.^178^ Similarly, YBX1 has been shown to stabilize mRNA by recruiting ELAVL1, which is another important m^5^C reader protein.^179^ In addition to affecting stability and export, YBX1 regulates translation of mRNAs. m^5^C methylation of IL-17A mRNA and intercellular adhesion molecule 1 (ICAM-1) mRNA promotes their translation.^180^^,^^181^ Moreover, NSUN2-mediated m^5^C methylation has also been shown to synergize with METTL3/METTL14-mediated m^6^A methylation to promote translation of p21 mRNA.^182^ However, m^5^C methylation catalyzed by NSUN2 had the opposite effect on p27 mRNA translation, suppressing its gene expression.^183^ Furthermore, m^5^C modifications are involved in regulation of mitochondrial function, stem cell development and differentiation, neurological development, cellular senescence, and other processes^184^^,^^185^^,^^186^ (Figure 1).

### Aberrant m^5^C RNA modifications in tumor immunity

m^5^C modification is associated with development of GC,^187^ bladder cancer,^179^ HCC,^188^ esophageal squamous cell carcinoma,^189^ and other tumors. In addition, in previous studies, the m^5^C demethylase TET has been proven to affect the function of immune cells.^190^^,^^191^^,^^192^ However, research on m^5^C in tumor immunity is still in its infancy. Previous studies have shown that NSUN2 methylates IL-17A mRNA and thereby increases IL-17 expression in T lymphocytes,^180^ and it can also increase leukocyte adhesion by methylating ICAM-1 mRNA.^181^ Regarding tumor immunity, the m^5^C reader YBX1 has been identified as a positive regulator of PD-L1 that induces immune evasion.^193^ Recent studies have shown that m^5^C RNA methylation regulators are prognostic predictors in certain cancers and can regulate immune cells in the tumor microenvironment. NSUN2 and NSUN6 have been identified as risk and protective factors, respectively, in triple-negative breast cancer (TNBC) and have been associated with six major immune cells, with the highest correlation between NSUN6 and CD4^+^ T cells.^194^ In another study, NSUN2 was found to negatively regulate immune cell infiltration, while high NSUN2 expression was associated with downregulation of multiple immune checkpoints.^195^ NSUN3 and NSUN4 have also been found to be related to immune cells in lung squamous cell carcinoma (LUSC); notably, NSUN3 was closely associated with CD8^+^ T cell infiltration, and NSUN4 was closely associated with neutrophils.^196^ m^5^C modifications may enhance humoral immunity, and in HNSCC, DNMT1 expression levels promote peptide cross-linking.^197^ In addition, the m^5^C eraser TET2 has also been shown to be significantly associated with infiltration of many immune cells. In papillary thyroid carcinoma,^198^ oral squamous cell carcinoma,^199^ and renal cell carcinoma,^200^ m^5^C scores were found to correlate with different immune phenotypes. A high m^5^C score (associated with an immunosuppressive phenotype) predicted a poor prognosis, while a low m^5^C score (associated with an immune-activating phenotype) predicted a good prognosis. Furthermore, in a study in prostate cancer, naive B cells, CD8^+^ T cells, M1 macrophages, and M2 macrophages were identified as key immune cells involved in distinguishing m^5^C-related immune phenotypes, and CTLA4 was differentially expressed between the two phenotypes.^201^

## Discussion

In this review, we focused on the relationship between m^6^A, m^1^A, and m^5^C modifications and tumor immunity and outlined different mechanisms by which RNA modifications affect antitumor immunity. Although RNA has over 100 modifications, the majority of studies have focused on m^6^A. In this review, we not only discussed m^6^A extensively but also focused on m^1^A and m^5^C, which have differences and similarities with m^6^A. On the one hand, these RNA modifications are all dependent on regulation of writers, erasers, and readers. In the presence of these regulators, RNA modifications are reversible and are precisely regulated to perform biological functions. All modifications are able to regulate the stability and translational efficiency of target RNAs. On the other hand, most of the more than 170 RNA modifications identified so far have been identified to occur on ncRNAs.^202^ m^6^A modifications are mainly found on mRNAs, whereas m^1^A and m^5^C modifications are mainly found on tRNAs and rRNAs^203^ and are present at much lower levels on mRNAs.^204^ While numerous studies have revealed the role played by m^6^A in tumor immunity, studies of m^1^A and m^5^C are still in the initial stages, and more investigation is needed. Recent studies have demonstrated the crucial roles played by m^1^A and m^5^C in tumorigenesis and immunity. For instance, the m^5^C modification at position 34 on mitochondrial tRNA^Met^ promotes metastasis by driving mitochondrial mRNA translation, increasing mitochondrial function, and affecting metabolic plasticity in tumors,^205^ indicating a pivotal function of m^5^C in tumorigenesis. TRMT61A mediates tRNA-m^1^A58 modification and is crucial for rapid T cell activation because it enhances the translation efficiency of Myc mRNA,^152^ indicating an essential role of m^1^A in regulating immunity. Therefore, it has been speculated that m^1^A modification is a vital regulator of tumor immunity. More studies should be carried out to determine the exact function of m^1^A and m^5^C in tumor immunity.

In addition, other types of RNA modifications have also been found to play a key role in regulation of tumor immunity. For example, the immunomodulatory function of A-to-I editing, which is catalyzed by adenosine deaminases acting on RNA (ADARs), has been described in previous studies.^206^ Recently, a new mechanism underlying the cytotoxicity of 6-thioguanine (6TG), a widely used chemotherapeutic agent, was found.^207^ 6TG increased A-to-I editing in bladder cancer-associated protein (BLCAP) mRNA, eventually decreasing cell viability.^207^ Moreover, knockdown of ADAR1 in tumor cells was found to enhance the response to ICIs by overcoming inactivation of antigen presentation.^208^ In addition to A-to-I editing, m^7^G modification of tRNA has recently been found to promote formation of an immunosuppressive microenvironment in HCC tumors mediated via the METTL1-transforming growth factor β2 (TGF-β2)-MDSC axis.^209^ Further exploration focusing on other types of RNA modifications is also needed.

Notably, increasing evidence suggests the potential for cross-talk between different RNA modifications. m^6^A, m^1^A, m^5^C, and other RNA modifications, including A-to-I editing and m^7^G^210^ and pseudouridine (Ψ)^211^ modification, have been found to modify mRNA. The cross-talk of mRNA modifications includes interactions of content and function. First, the abundance of RNA modifications on mRNA is modulated by other modifications. For instance, there is a negative correlation between m6A and A-to-I editing, two of the most abundant RNA modifications affecting mRNA, and both occur at adenosine.^212^ Second, different modifications on mRNA cooperate in regulating the fate of mRNA. For example, NSUN2-mediated m^5^C methylation promotes METTL3/METTL14-mediated m^6^A methylation in p21 mRNA and enhances p21 expression and vice versa.^182^ HRSP12 binds to m^1^A and promotes the interaction between m^6^A modifications and YTHDF2, thus cooperatively promoting rapid mRNA degradation.^213^ Additionally, modifications of ncRNAs can also cross-talk with modifications of mRNAs. For example, the tRNA methyltransferase TRMT10A not only installs N1-methylguanosine (m^1^G) on tRNA but also facilitates the m^6^A demethylase activity of FTO and potentially accelerates degradation of mRNA in a YTHDF2-dependent manner.^214^ However, the precise coregulation networks involving different RNA modifications and the impact of these interactions on tumor immunity remain largely unclear; these topics are promising areas for further research.

Furthermore, studies have revealed that different epigenetic modifications, including DNA methylation, chromatin remodeling, histone modification, and RNA modification, do not function independent of each other and share interaction networks.^215^ For example, m^6^A modification of CBX1 (a histone methylation regulator) mRNA destabilized the transcript via the m^6^A reader YTHDF3.^113^ In addition, METTL3/METTL14 downregulates H3K9me2 modification by YTHDC1-mediated recruitment of the H3K9me2 demethylase KDM3B to m^6^A-associated chromatin regions, which indicates that there is direct information flow from RNA to chromatin.^71^ On the other hand, m^6^A modification of mRNA can be regulated by histone modifications.^216^ For example, histone H3 trimethylation at Lys36 (H3K36me3) can be directly recognized by METTL14 and promote m^6^A modification of nascent RNAs to coregulate transcription, with MTC interacting with adjacent RNA polymerase II molecules.^217^ Recent studies have shown a similar regulatory mechanism between DNA methylation and RNA modifications. For instance, RNA m^6^A guides DNA 5-methylcytosine (5mC) demethylation via the m^6^A reader FXR1, recruiting TET1 to genomic loci and reprogramming chromatin accessibility.^218^ However, the precise cross-talk between different RNA modifications that regulate the antitumor immune response needs to be further explored and verified.

In summary, RNA modifications are closely associated with tumor immunity and may be novel targets for preventing immune escape. The regulation of oncogenic signaling pathways by RNA modifications revealed in previous studies has identified potential drug targets, such as the Wnt/β-catenin pathway, PI3K-Akt-mTOR pathway, and JAK-STAT pathway.^219^ In this review, we examined RNA modifications and tumor immunity, offering ideas for therapy for many patients who have shown poor responses to existing immunotherapies. Inhibitors of ALKBH5/FTO and targeted deletion of METTL3/14 have been shown to enhance the effects of PD-L1 inhibitor immunotherapy by enhancing the expression of immune checkpoints and secretion of cytokines.^112118121^ In addition, RNA-modified regulators hold promise for development as biological indicators of cancer prognosis and response to immune checkpoint blockade. Further clinical studies are urgently needed. However, current applications generally remain limited by the lack of relevant small-molecule inhibitors and unknown mechanisms of action. A deeper understanding of the biological mechanisms is conducive to selection of more efficient targets and drugs. These outcomes might further improve the survival time of cancer patients and their quality of life.

## Lead contact website

https://www.shsmu.edu.cn/info/1026/5530.htm

http://daoshi.shsmu.edu.cn/Pages/TeacherInformationView.aspx?uid=3823333C-AD6F-44C4-9118-C632E793BCBD&from=s&pId=100212&tId=
